# Anti-tumor necrosis factor-alpha inhibitor-induced linear psoriasis: A case report

**DOI:** 10.1177/2050313X241304954

**Published:** 2024-12-11

**Authors:** Olivia C. MacIntyre, Kerri Purdy

**Affiliations:** 1 Faculty of Medicine, Dalhousie University, Halifax, NS, Canada; 2 Division of Clinical Dermatology and Cutaneous Science, Department of Medicine, Dalhousie University, Halifax, NS, Canada

**Keywords:** Linear psoriasis, Blaschkoid psoriasis, anti-tumor necrosis factor-alpha inhibitor, infliximab, Enstilar, hidradenitis suppurativa

## Abstract

A 56-year-old male presented to the clinic for follow-up of severe, longstanding hidradenitis suppurativa. On physical examination, there was a linear Blaschkoid distribution of erythematous scaly papules extending from the left upper arm toward the scapular mid-back region. A clinical diagnosis of linear psoriasis was made, and the patient’s dosage interval of infliximab was decreased to every 6 weeks. The patient was offered Betamethasone diproprionate/calcipotriol (Enstilar) topical foam to use daily which demonstrated efficacy in clearing 90% of the affected areas. This case illustrates the potential for Betamethasone diproprionate/calcipotriol (Enstilar) topical foam use as a treatment approach for anti-tumor necrosis factor-alpha inhibitor-induced linear psoriasis. At present there is no consensus on linear psoriasis pathogenesis or best treatment approach, therefore, more research is needed to understand its pathogenesis and establish management guidelines.

## Introduction

The concept of anti-TNF-α inhibitor use introducing paradoxical psoriasis in patients is not a novel phenomenon.^1–3^ Nonetheless, there are few reports detailing the development of linear psoriasis as a result of anti-TNF-α inhibitor use in the literature.^4^ Linear psoriasis is a rare presentation of psoriasis, characterized by erythematous plaques and/or papules following the lines of Blaschko in the absence of mid-line traversal.^5^ Given its rarity, uncertainty remains regarding its pathogenesis. Further, treatment can be challenging, as guidelines for linear psoriasis management have yet to be established. To aid in remedying this discrepancy, we present a case of linear psoriasis having developed following long-term use of anti-TNF inhibitors for a patient with hidradenitis suppurativa (HS), and its successful treatment using topical Enstilar foam.

## Case report

**Figure 1.**
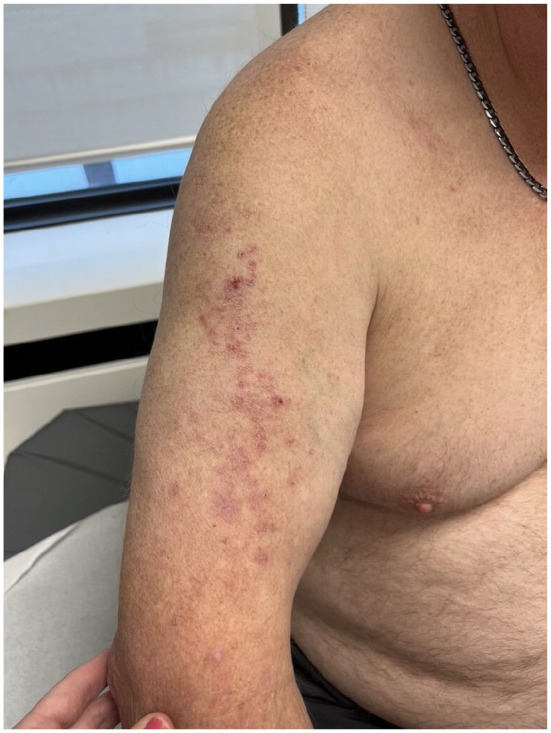
Psoriasiform eruption in a linear distribution on the right arm following anti-TNF-alpha inhibitor use.

A 56-year-old male presented to the clinic for follow-up of severe, longstanding HS. At the time, the patient had been on a 2-year course of infliximab 10 mg kg^−1^ every 4 weeks for treatment of HS. The patient described a new psoriasiform eruption on his right arm and back. On physical examination, there was a linear Blaschkoid distribution of erythematous scaly papules extending from the right upper arm toward the scapular mid-back region (Figure 1). There were no psoriatic lesions elsewhere on the body, and the patient reported an absence of trauma to the area. No personal or family history of psoriasis was disclosed. A clinical diagnosis of linear psoriasis was made, and the patient was initiated on topical therapy.

**Figure 2.**
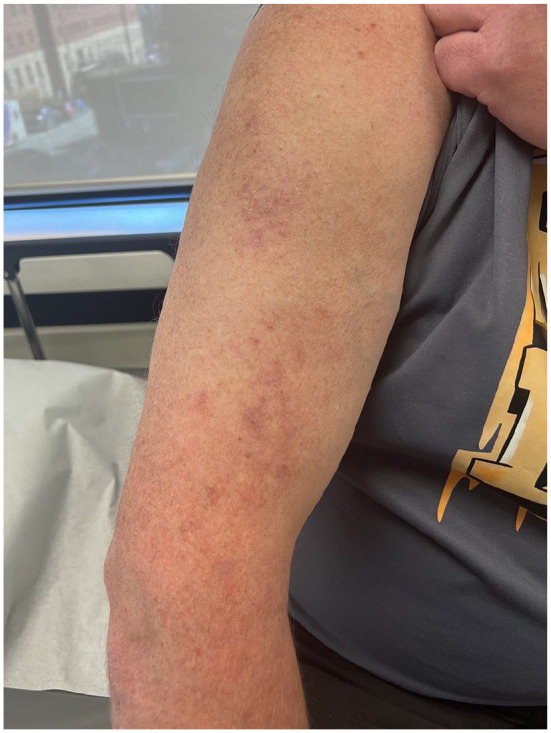
Post-inflammatory dyspigmentation following treatment with topical Enstilar foam.

Treatment with Betaderm 0.1% ointment was initiated twice daily to the affected area but was not effective. The patient was also interested in decreasing the dose of infliximab to see if that would be of benefit, so the interval was changed to every 6 weeks. The patient was then offered Betamethasone diproprionate/calcipotriol (Enstilar) topical foam to use daily and this was effective at clearing 90% of the affected areas (Figure 2).

## Discussion

Linear psoriasis is a rare variant of psoriasis, with few cases reporting its development independent of concurrent classical psoriasis presentations.^6–8^ First described in 1951, linear psoriasis lesions are thought to result from an interaction between genetic mosaicism and environmental factors.^9^ Linear psoriasis may be mistaken clinically for inflammatory linear verrucous epidermal nevus (ILVEN). However, ILVEN often develops in a slow, progressive manner from birth, contrasting linear psoriasis’s rapid development in later stages of life. Further, ILVEN lesions are predominantly pruritic and unresponsive to traditional psoriasis therapies.^10^ Nonetheless, these conditions can often be visibly mistaken for one another, which may contribute to the underrepresentation of linear psoriasis in the literature. Given our patient’s characteristic clinical presentation and resolution of the lesions with topical steroid/vitamin D analog, the diagnosis of linear psoriasis was able to be made without histological examination.

In a large-scale study that retrospectively analyzed various linear psoriasis presentations, the concept of anti-TNF-α inhibitor use unmasking linear psoriasis was discussed. Of the 30 case studies that were presented, 2 patients had developed linear psoriasis following treatment with etanercept and infliximab, respectively, after 2 months of treatment.^5^ In addition, a cohort study of over 7000 patients identified infliximab as having the highest risk for new-onset psoriasis when compared to patients on etanercept and adalimumab as well as the general population.^1^

While the factors contributing to linear versus classical psoriasis development in those using anti-TNF-α inhibitor therapies remains unclear, we present a case to add to the literature of a patient with HS that developed linear psoriasis in a Blaschkoid distribution felt to be induced by treatment with infliximab. At present, there is no consensus on linear psoriasis pathogenesis or the best treatment approach. However, the use of topical Enstilar foam demonstrated efficacy in clearing 90% of the affected areas in our case, giving merit to its potential use. Currently, there is no way to determine if a patient is at risk for the development of linear psoriasis while on anti-TNFF-α inhibitor therapy; perhaps these questions will be satisfactorily answered with future research.
